# Involvement of abscisic acid, ABI5, and PPC2 in plant acclimation to low CO_2_

**DOI:** 10.1093/jxb/eraa148

**Published:** 2020-03-24

**Authors:** Lei You, Jumei Zhang, Long Li, Chuanlei Xiao, Xinhua Feng, Shaoping Chen, Liang Guo, Honghong Hu

**Affiliations:** 1 National Key Laboratory of Crop Genetic Improvement, Huazhong Agricultural University, Wuhan, China; 2 University of Birmingham, UK

**Keywords:** Abscisic acid, *Arabidopsis thaliana*, ABI5, carbon–nitrogen balance, low CO_2_, PEPC, photorespiration, photosynthesis

## Abstract

Phosphoenolpyruvate carboxylase (PEPC) plays a pivotal role in the photosynthetic CO_2_ fixation of C_4_ plants. However, the functions of PEPCs in C_3_ plants are less well characterized, particularly in relation to low atmospheric CO_2_ levels. Of the four genes encoding PEPC in Arabidopsis, *PPC2* is considered as the major leaf PEPC gene. Here we show that the *ppc2* mutants suffered a growth arrest when transferred to low atmospheric CO_2_ conditions, together with decreases in the maximum efficiency of PSII (*F*_v_/*F*_m_) and lower levels of leaf abscisic acid (ABA) and carbohydrates. The application of sucrose, malate, or ABA greatly rescued the growth of *ppc2* lines under low CO_2_ conditions. Metabolite profiling analysis revealed that the levels of glycine and serine were increased in *ppc2* leaves, while the abundance of photosynthetic metabolites was decreased under these conditions. The transcript levels of encoding enzymes involved in glycine or serine metabolism was decreased in *ppc2* in an ABI5-dependent manner. Like the *ppc2* mutants, *abi5-1* mutants had lower photosynthetic rates and *F*_v_/*F*_m_ compared with the wild type under photorespiratory conditions (i.e. low CO_2_ availability). However, the growth of these mutants was similar to that of the wild type under non-photorespiratory (low O_2_) conditions. The constitutive expression of *ABI5* prevented the growth arrest of *ppc2* lines under low CO_2_ conditions. These findings demonstrate that PPC2 plays an important role in the acclimation of Arabidopsis plants to low CO_2_ availability by linking photorespiratory metabolism to primary metabolism, and that this is mediated, at least in part, through ABA- and ABI5-dependent processes.

## Teaser

PPC2, together with ABI5 and ABA, is involved in plant acclimation to low CO_2_ through linking photorespiratory metabolism with primary metabolism.

## Introduction

Phosphoenolpyruvate carboxylase (PEPC; EC 4.1.1.31) is a ubiquitous enzyme in plants, algae, and bacteria (Chollet *et al.*, 1996). There are two functional forms of PEPC in higher plants, namely the photosynthetic and non-photosynthetic isoforms. In crassulacean acid metabolism (CAM) and C_4_ plants, PEPC plays a pivotal photosynthetic role in primary CO_2_ fixation, by catalyzing β-carboxylation of PEP with HCO_3_^−^ to oxaloacetate and inorganic phosphate. Photosynthetic PEPC activity in C_4_ plants is essential for carbon assimilation via pre-fixation of CO_2_ in the bundle sheath, and thus decreases photorespiratory activity and contributes to greater water use efficiency and photosynthetic efficiency compared with C_3_ plants (Taylor *et al.*, 2018). The non-photosynthetic PEPCs play key roles in plant primary metabolism by replenishing the tricarboxylic acid (TCA) cycle to support carbon and nitrogen metabolism (Masumoto *et al.*, 2010). Moreover, they are also important for stomatal opening (Gehlen *et al.*, 1996) and supplying malate as a respiration substrate to symbiotic N_2_-fixing bacteroids in legume root nodules (Vidal and Chollet, 1997). PEPCs in C_3_ plants are believed to play minor roles in photosynthesis or photorespiration (von Caemmerer, 2013). Recently, ^13^C fixation NMR analysis in sunflower indicated that PEPC activity significantly increased along with decreases in net CO_2_ assimilation under high photorespiratory conditions (Abadie *et al.*, 2017; Abadie and Tcherkez, 2019). Moreover, *OsPPC4* mutation in rice led to great accumulation of photorespiratory intermediates such as glycine, serine, and glycerate (Masumoto *et al.*, 2010). Since photorespiration is closely related to primary metabolism (Mouillon *et al.*, 1999; Rachmilevitch *et al.*, 2004), these studies imply that C_3_ PEPCs may play certain roles in photosynthesis and photorespiration.

In Arabidopsis, there are four genes encoding PEPC. Mutations in either *PPC1*, *PPC2*, or *PPC3* lead to decreases in fresh weight and flowering time delay (Feria *et al.*, 2016). Moreover, *PPC1* and *PPC2* mutations greatly reduced malate and citrate synthesis, and severely suppressed ammonium assimilation, which finally leads to *ppc1ppc2* growth arrest (Shi *et al.*, 2015). However, little is known about how these *PPC* genes regulate plant primary metabolism, and whether they regulate photosynthesis and plant development under stress conditions such as low CO_2_ and drought stress.

Abscisic acid (ABA) plays a prominent role in plant stress tolerance, and regulates various important plant development aspects, such as seed dormancy, germination, seedling establishment (Finkelstein *et al.*, 2002), and vegetative development (Sharp, 2002). ABA regulates these processes through affecting gene expression by modulating ABA-responsive transcription factors, such as B3-domain family proteins (e.g. ABI3 and VAL1) (Giraudat *et al.*, 1992; Suzuki *et al.*, 2007), APETALA2 (AP2) family proteins (e.g. ABI4) (Finkelstein *et al.*, 1998), and basic leucine zipper (bZIP) family proteins (e.g. ABI5) (Finkelstein and Lynch, 2000). ABI5 is a key component in ABA-triggered pathways during germination, seedling establishment, and vegetative growth (Lopez-Molina *et al.*, 2001); in addition, it has been reported to play certain roles in nitrogen assimilation and signaling (Signora *et al.*, 2001). ABI5 also positively regulates *SGR1* and *NYC1*, two chlorophyll catabolism-related genes, through recognizing their upstream ABA-responsive elements (ABREs) (Sakuraba *et al.*, 2014). Hence, ABI5 has been proposed as a key regulator to monitor environmental conditions during seedling growth. However, it remains unknown whether ABI5 is responsive to CO_2_ changes and affects plant growth under different CO_2_ conditions.

Environmental changes have a rapid effect on plant intercellular CO_2_ concentration (*C*_i_). Plant leaf photosynthesis under light conditions causes a quick drop of *C*_i_ to <200 ppm (Engineer *et al.*, 2016). In addition, drought stress triggers stomatal closure, thereby reducing CO_2_ uptake and lowering *C*_i_, which results in reduced photosynthesis (Parry *et al.*, 2013). In this study, we demonstrated the crucial role of *AtPPC2* in seedling growth under low CO_2_ conditions by linking photorespiration metabolism with primary metabolism, with the involvement of ABI5. *ppc2* mutants showed retarded seedling growth, reduced CO_2_ assimilation, and suppressed *ABI5* expression under low CO_2_ conditions. Metabolic analysis showed the accumulation of the photorespiratory intermediates glycine and serine, and a decrease of malate in *ppc2* under low CO_2_ conditions. *ABI5* overexpression rescued the growth arrest phenotype at low CO_2_. Taken together, our results demonstrate that *PPC2* and *ABI5* are key regulators of plant acclimation to low CO_2_, and positively contribute to carbon fixation and metabolism in C_3_ plants.

## Materials and methods

### Plant materials and growth conditions

All *ppc* mutant lines used in this study were in the Columbia (Col-0) background. The mutant lines *ppc1* (SALK_088836) (Feria *et al.*, 2016), *ppc2* (SALK_128516) (Shi *et al.*, 2015), and *ppc3* (SALK_143289) (Feria *et al.*, 2016) were obtained from the Arabidopsis Biological Resource Center (http://abrc.osu.edu), and their homozygosity was confirmed by PCR (Supplementary Table S1 at *JXB* online).

Seeds were surface-sterilized and germinated. For *A*–*C*_i_ curves and drought stresses, plants were grown in pots and for all other experiments the plants were in Petri dishes. All plants in pots and Petri dishes were grown in CO_2_-controlled growth chambers (Percival), in which CO_2_ concentrations can be accurately and stably controlled in the range of 100–1500 ppm, with a light regime of 16 h light/8 h dark (light intensity 100 μmol m^−2^ s^−1^) and a relative humidity of 56%. For mild drought stress, a total of 64 five-day-old seedlings were transferred into a new pot and grown for 5 d and then the plants were not watered for 10 d.

### Generation of constructs and transgenic plants

The coding sequences (CDSs) of *PPC2* and *ABI5* were amplified from Arabidopsis cDNA with primers PPC2-OE-F/PPC2-OE-R and ABI5-OE-F/ABI5-OE-R, respectively. The PCR products were cloned into pGreen-35S and pEarlyGate-35S-YFP (Earley *et al.*, 2006). The *PPC2* promoter region amplified by primers Pro-PPC2-F and Pro-PPC2-R was cloned into the pEarlyGate-100-GUS vector (Earley *et al.*, 2006). All primers used for generation of construct are presented in Supplementary Table S1. The constructs were introduced into Arabidopsis by *Agrobacterium tumefaciens*-mediated transformation using the floral dipping method (Clough and Bent, 1998).

### Determination of the chlorosis rate

Fifteen-day-old seedlings of Col-0 and the *ppc2* mutant grown at 200 ppm and 400 ppm CO_2_ were analyzed for the chlorosis rate. Cotyledons which were almost bleached were defined as chlorotic cotyledons. The chlorosis rate was determined as:

$$\begin{array}{ll} Chlorosis\;rate\left(\%\right)= & \;\;number\;of\;chlorotic\;seedlings/\\ & \;\;number\;of\;total\;seedlings \end{array}$$

### PEPC activity assays

Proteins were extracted from leaves (0.1 g FW) of 15-day-old seedlings in 1 ml of extraction buffer [200 mM HEPES-NaOH (pH 7), 10 mM MgCl_2_, 5 mM DTT, and 2% (w/v) polyvinylpyrrolidone-40]. After centrifugation, the supernatant was immediately used for PEPC activity detection by a PEPC activity assay kit (Jiancheng Bioengineering Institute, Nanjing, China). One unit of PEPC activity was defined as 1 nmol of NADH oxidation per min per mg protein at 25 °C. The total protein content was determined by using a BCA (bicinchoninic acid) protein assay kit (Sangon Biotech).

### *A*–*C*_i_ curve analysis

The measurements of *A*–*C*_i_ curves were performed by a closed infrared gas exchange analysis system (LI-COR 6400XT). Four- to five-week-old leaves were clamped in the 2 cm^2^ chamber with leaf temperature at 21 °C for measurement. The *A*–*C*_i_ curve measurements were performed at CO_2_ concentrations of 50, 100, 200, 300, 400, 600, and 800 ppm with a photosynthetic photon flux density of 2000 μmol m^−2^ s^−1^. The relative humidity was ~50% in all measurements. *A*–*C*_i_ curves under low oxygen conditions were performed by replacing air with pure N_2_.

### Analyses of sucrose and starch contents

Sucrose and starch were extracted from 15-day-old seedlings and estimated by using the Sucrose Colorimetric/Spectrophotometric Assay kit (Comin Biotechnology Co., Ltd, Suzhou, China) and Starch Colorimetric/Spectrophotometric Assay kit (Comin Biotechnology Co., Ltd) according to the manufacturer’s instructions.

### Analyses of chlorophyll content and chlorophyll fluorescence

Total leaf chlorophyll was extracted with 80% acetone at 4 °C for 24 h in darkness, and then the supernatant was used for the absorbance measurement with a spectrophotometer (BeckMan Coulter DU730). The total chlorophyll content was calculated with the following formula:

$$\begin{array}{ll} Total\;chlorophyll\;\left(nmol\;ml^{1}\right)= & \;\;19.54\times \left(A_{646.8}A_{720}\right)\\ & \;+8.29\times \left(A_{663.2}A_{720}\right) \end{array}$$

Chlorophyll fluorescence of 15-day-old seedlings was analyzed by using a FluorCam PAM as described in a previous report (Baker, 2008).

### Endogenous ABA quantification

Crude extracts were prepared from leaves (0.1 g FW) of 15-day-old seedlings grown at 200 ppm and 400 ppm in 750 μl of 80:19:1 methanol:H_2_O:acetic acid buffer supplemented with internal standards for 6 h. After centrifugation, the extracts were filtered through a 0.22 μm filter and dried with N_2_ at room temperature, and then dissolved in 200 μl of methanol. ABA quantification was performed as previously described (Liu *et al.*, 2012).

### Semi-quantitative PCR and quantitative real-time PCR

Total RNA was extracted from whole seedlings or leaves using TRIzol reagent (Invitrogen Life Technologies), according to the manufacturer’s instructions. The cDNA was reverse transcribed from 1 μg of total RNA using M-MLV reverse transcriptase (Promega) in a reaction buffer [50 mM Tris–HCl (pH 8), 75 mM KCl, 3 mM MgCl_2_, 10 mM DTT, 0.5 mM dNTP, and 0.5 μg oligo(dT)_15_]. Quantitative RT-PCR was performed by using a Universal SYBR^®^ Green kit and the C1000 Touch Thermal Cycler real-time PCR detection system (Bio-Rad). The *EF1α* (AT5G60390) gene was used as a reference for mRNA normalization. The comparative cycle threshold (C_t_) method was used to evaluate the relative gene expression levels. The primers used for the expression analysis are listed in Supplementary Table S1.

### GUS histochemical analysis

β-Glucuronidase (GUS) histochemical analysis was carried out on transgenic lines expressing *ProPPC2*::GUS. Plants at various stages including emerging seedling, 7-day-old seedling, 15-day-old seedling, flowers, and siliques were stained in a GUS staining solution and imaged by a Nikon microscope.

### Quantification of amino and organic acids

Crude extracts for amino acid determination were prepared from leaves (0.2 g FW) of 15-day-old seedlings in 8% (w/v) 5-sulfosalicylic acid for 1 h followed by centrifugation. The supernatants were filtered through a 0.22 μm filter. The amino acid contents were determined by LC-MS/MS with an Agilent 1290 Infinity II and Agilent 6460 (Kowalski *et al.*, 2017).

For organic acid determination, crude extracts were obtained from shoots (0.2 g FW) of 15-day-old seedlings grown at 200 ppm and 400 ppm. The samples were homogenized in 3 ml of 7:3 methanol:chloroform (–20 °C) for 2 h. The water-soluble metabolites were extracted from the chloroform phase by the addition of 2.4 ml of H_2_O. After shaking and centrifugation, the upper methanol–H_2_O phase was transferred and dried with N_2_ at room temperature. The extracts were dissolved in 200 μl of H_2_O and transferred to 0.45 μm cellulose acetate centrifuge tube filters. The determination of organic acid contents was performed by an AB SCIEX QTRAP 6500 Plus LC-MS/MS system as previously described (Ma *et al.*, 2014).

### Luciferase assay

Arabidopsis protoplasts were isolated from 4- to 6-week-old plants following the method used in a previous report (Yoo *et al.*, 2007). Plasmid DNA (15 µg) was used for polyethylene glycol-calcium transformation (PEG4000), and the protoplast transformation was performed as previously described (Yoo *et al.*, 2007).

Cellular extracts of Arabidopsis protoplasts after transformation with different constructs were collected for dual-luciferase assays (Hellens *et al.*, 2005). Cellular extracts (30 μl) were used to detect the firefly and Renilla luciferase activities by a Mithras LB 940 Multimode Microplate Reader. Luciferase activity was normalized to the Renilla activity. All experiments were performed at least three times.

### Nuclear and cytoplasmic protein extraction

Arabidopsis protoplasts were collected and homogenized in 100 μl of lysis buffer. After centrifugation, the supernatant for cytoplasmic protein extraction was boiled in SDS–PAGE loading buffer for 2 min. Insoluble nuclei for nuclear protein extraction were re-suspended in 1.5 ml of nuclei resuspension buffer with 0.2% Triton X-100 and then centrifuged. The resuspension was repeated three times (Xu *et al.*, 2012).

### Accession numbers

The gene accession numbers in this study are available at TAIR (The Arabidopsis Information Resource): *PPC1* (AT1G53310), *PPC2* (AT2G42600), *PPC3* (AT3G14940), *ABI3* (AT3G24650), *ABI4* (AT2G40220), *ABI5* (AT2G36270), *GGAT1* (AT1g23310), *GGAT2* (AT1g70580), *SGAT1* (AT2G13360), *GLDT1* (AT1G11860), *GLDP1* (At4g33010), and *SHMT1* (At4g37930).

## Results

### The *ppc2* mutant showed growth arrest under low CO_2_ conditions

**Fig. 1.**
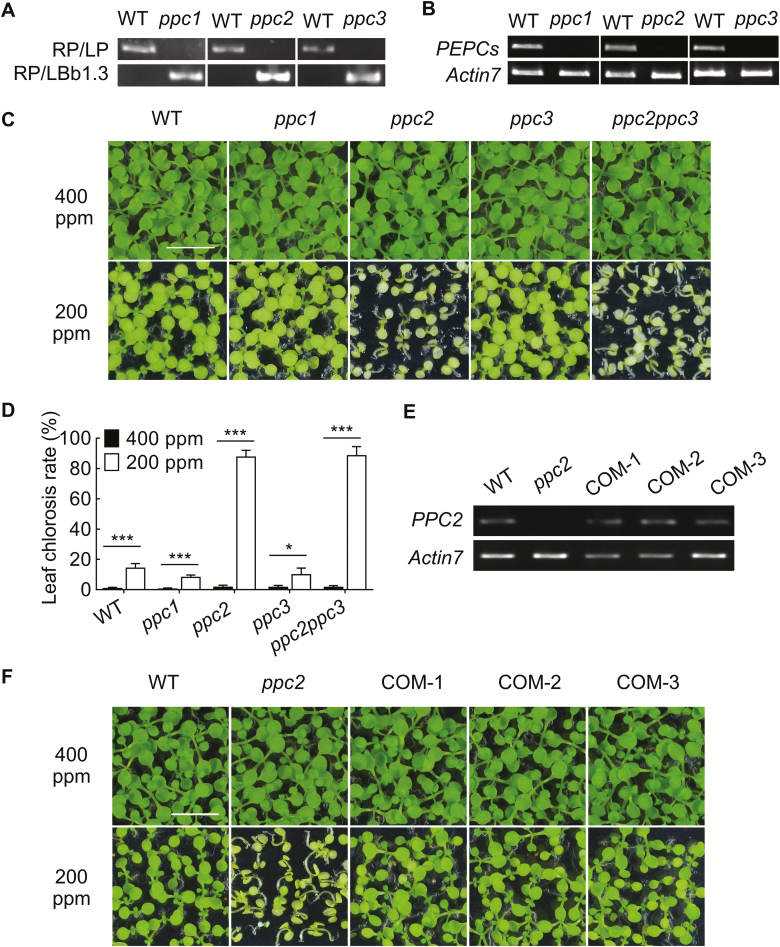
Growth arrest phenotype of *ppc2* mutant seedlings under low CO_2_ conditions. (A) Genotyping and (B) expression analysis of *PPC1*, *PPC2*, and *PPC3* in their corresponding single mutants of *ppc1*, *ppc2,* and *ppc3*. *ACTIN7* (AT5G09810) was used as a control. (C) Phenotype and (D) statistical analysis of the chlorosis rate of wild-type (WT), *ppc1*, *ppc2*, *ppc3,* and *ppc2ppc3* seedlings grown on sucrose-free 1/2 MS medium at 400 ppm or 200 ppm CO_2_ for 15 d. Data shown are mean ±SEM (*n*=4). Each replicate has at least 60 seedlings. Asterisks indicate significant differences between genotypes (*P<0.05; ***P<0.005 by Student’s *t*-test; ns, no significant difference). (E) Expression levels of *PPC2* in *PPC2-*overexpressing *ppc2* plants determined by RT-PCR. RNA was extracted from leaves of 15-day-old seedlings. *ACTIN7* (AT5G09810) was used as a control. (F) Growth phenotypes of seedlings of the WT, *ppc2,* and *PPC2* complementary lines (COM-1, COM-2, and COM-3) grown on sucrose-free 1/2 MS medium at 400 ppm or 200 ppm CO_2_ for 15 d. Each replicate has at least 60 seedlings. Scale bars=1 cm in (C) and (F).

To determine whether the Arabidopsis PEPCs are involved in plant growth regulation under low CO_2_ conditions, we evaluated the growth performance of T-DNA insertion lines of plant-type PEPCs, *ppc1* (Salk_088836) (Feria *et al.*, 2016), *ppc2* (Salk_128516) (Shi *et al.*, 2015), and *ppc3* (Salk_143289) (Feria *et al.*, 2016), on sucrose-free half-strength Murashige and Skoog (1/2 MS) medium under low CO_2_ (200 ppm) conditions for 15 d, as well as the control ambient CO_2_ condition (400 ppm). These single mutants were determined as knockout mutants by our analysis (Fig. 1A, B) and previous studies (Shi *et al.*, 2015; Feria *et al.*, 2016). Under ambient CO_2_ conditions, no obvious morphological differences were observed among the *ppc* and Col-0 seedlings (Fig. 1C). Under low CO_2_ conditions, the cotyledons of Col-0, *ppc1*, and *ppc3* turned pale green without obvious morphological differences among them. Interestingly, *ppc2* mutant plants exhibited a smaller size and chlorosis when compared with Col-0, *ppc1*, and *ppc3* (Fig. 1C, D). The chlorotic rate was 87.5% in *ppc2*, and only 10–20% in Col-0, *ppc1*, and *ppc3* (Fig. 1D). These results suggest that *PPC2* is required for seedling growth under low CO_2_ conditions.

To determine whether the growth arrest of *ppc2* was due to a defect in seed germination, we assessed the seed germination rates of *ppc* and Col-0 seeds under low CO_2_ conditions on sucrose-free 1/2 MS medium. Similar germination rates and status were observed among Col-0, *ppc1*, *ppc2*, and *ppc3* (Supplementary Fig. S1), revealing that the *ppc2* growth arrest phenotype occurred after germination, and *PPC2* is involved in seedling development under low CO_2_ conditions. To determine whether *PPC1* or *PPC3* has effects on seedling growth under low CO_2_ conditions in a *PPC2-*dependent manner, we crossed the *ppc1* and *ppc3* mutants with *ppc2*. *ppc2ppc3* and *ppc1ppc2* double mutants were obtained, but we could not obtain the *ppc1ppc2* double mutant seeds due to severe growth arrest, consistent with a previous study (Shi *et al.*, 2015). The *ppc2ppc3* double mutant showed a similar growth phenotype and seed germination to the *ppc2* single mutant under low CO_2_ conditions (Fig. 1C; Supplementary Fig. S1), suggesting that *PPC3* possibly is not essential for seedling growth under low CO_2_ conditions.

To confirm that *PPC2* is responsible for the growth retardation observed in *ppc2*, the *PPC2* CDS driven by the *Cauliflower mosaic virus* (CaMV) 35S promoter was introduced into the *ppc2* mutant. The growth arrest and cotyledon chlorotic phenotypes were all rescued in the lines by *PPC2* expression (Fig. 1E, F). These results demonstrate that *PPC2* is the causal gene, and is a major regulator of seedling growth under low CO_2_ conditions.

### *PPC2* encodes a major PEPC in Arabidopsis leaves

**Fig. 2.**
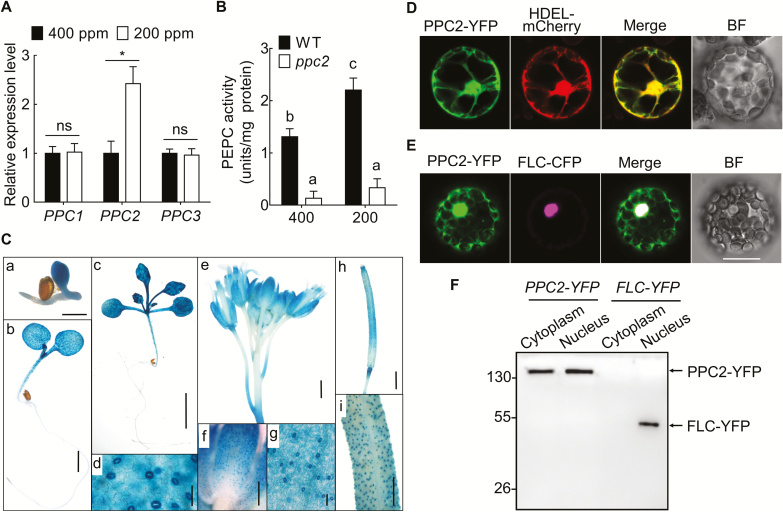
*PPC2* is low CO_2_ inducible and encodes a major PEPC in Arabidopsis leaves. (A) Expression levels of *PPC1*, *PPC2*, and *PPC3* in 15-day-old seedlings of Col-0 grown on sucrose-free 1/2 MS medium at 400 ppm or 200 ppm CO_2_. Expression levels are expressed relative to that of *EF1α* (AT5G60390). Data shown are mean ±SEM (*n*=3). Asterisks indicate significant differences between genotypes (*P<0.05; ns, no significant difference). (B) Total leaf PEPC activity in 15-day-old wild-type and *ppc2* seedlings under ambient and low CO_2_ conditions. Data shown are mean ±SEM (*n*=3). Different letters indicate significant differences using Tukey’s test at *P*≤0.05. (C) Tissue-specific expression of *PPC2* in leaves, flowers, and siliques. a–c, GUS activity in leaves of 3-day-old (a), 5-day-old (b), and 15-day-old (c) seedlings; d, guard cells of cotyledon; e and f, GUS activity in calyxes; g, guard cells of calyx; h, GUS activity in siliques; i, guard cells of siliques. Scale bars=50 μm in d and g, and 1 mm in a–c, e, f, h, and i. (D, E) Subcellular localization of PPC2–YFP co-expressed with the ER marker HDEL–mCherry (D) and nuclear-localized FLC–YFP (E) in protoplasts from 4-week-old Col-0. BF, bright field. YFP, yellow fluorescent protein. Scale bar=20 μm. (F) Western blot analyses of PPC2–YFP and FLC–YFP levels in the cytoplasmic and nuclear fractions of Arabidopsis protoplasts expressing *PPC2-YFP* or *FLC-YFP*.

The expression levels of these three *PEPC* genes under low CO_2_ conditions were determined by qPCR analysis. Only *PPC2* was induced by low CO_2_ (Fig. 2A), consistent with a previous study (Li *et al.*, 2014). To confirm that PPC2 is a functional PEPC in Arabidopsis, we measured the total PEPC activity in *ppc2* and Col-0 under low and ambient CO_2_ conditions. The *ppc2* mutant lost most of its PEPC activity in leaves under both 400 ppm and 200 ppm CO_2_ conditions (Fig. 2B). Moreover, low CO_2_ treatment increased the PEPC activity in Col-0 but not in *ppc2* leaves (Fig. 2B). These results suggest that PPC2 is a major PEPC in Arabidopsis leaves and is responsive to low CO_2_.

We then determined the *PPC2* expression pattern by expressing *PPC2pro::GUS* in Col-0. GUS staining showed that *PPC2* expression was high in leaves, hypocotyls, flowers, and siliques, but low in roots (Fig. 2C). At the cellular level, *PPC2* was highly expressed in guard cells. The PPC2 subcellular localization was also determined by expressing *35S::PPC2-YFP* in Col-0 protoplasts. Yellow fluorescence protein (YFP) fluorescence revealed PPC2 localization in the cytoplasm, nucleus, and also the endoplasmic reticulum (ER; Fig. 2D), which was confirmed by western blot and co-expression with the ER marker HDEL (Fig. 2E, F). All these results demonstrate a specific role for *PPC2* in plant growth regulation under low CO_2_ conditions.

### *ppc2* seedlings showed reduced carbon assimilation under low CO_2_ conditions

To determine whether the growth arrest and cotyledon chlorosis of the *ppc2* mutant under low CO_2_ conditions are due to defects in carbohydrate accumulation, we detected the starch content by iodine staining and quantification in 15-day-old *ppc2* and Col-0 seedlings at the end of the illumination period (22.00 h) and darkness period (06.00 h) under different CO_2_ concentrations. Under ambient CO_2_ conditions, no obvious difference was found in starch accumulation between *ppc2* and Col-0 plants (Supplementary Fig. S2A, B). However, under low CO_2_ conditions, starch accumulation was significantly reduced in *ppc2* at both time points (Supplementary Fig. S2A, B). The sucrose content of *ppc2* and Col-0 under the same conditions was then measured. *ppc2* seedlings had reduced sucrose content under both low and ambient CO_2_ conditions compared with Col-0 (Supplementary Fig. S2B). Moreover, the synthetic substrates of starch and sucrose, such as G6P, F6P, G1P, ADPG, UDPG, and Suc6P, were also lower in *ppc2* under low CO_2_ conditions, but comparable with Col-0 under ambient CO_2_ conditions (Supplementary Fig. S2B), consistent with the starch and sucrose contents in *ppc2* (Supplementary Fig. S2A, B). These results suggested that *PPC2* mutation led to decreased photosynthetic carbohydrate accumulation under low CO_2_ conditions.

**Fig. 3.**
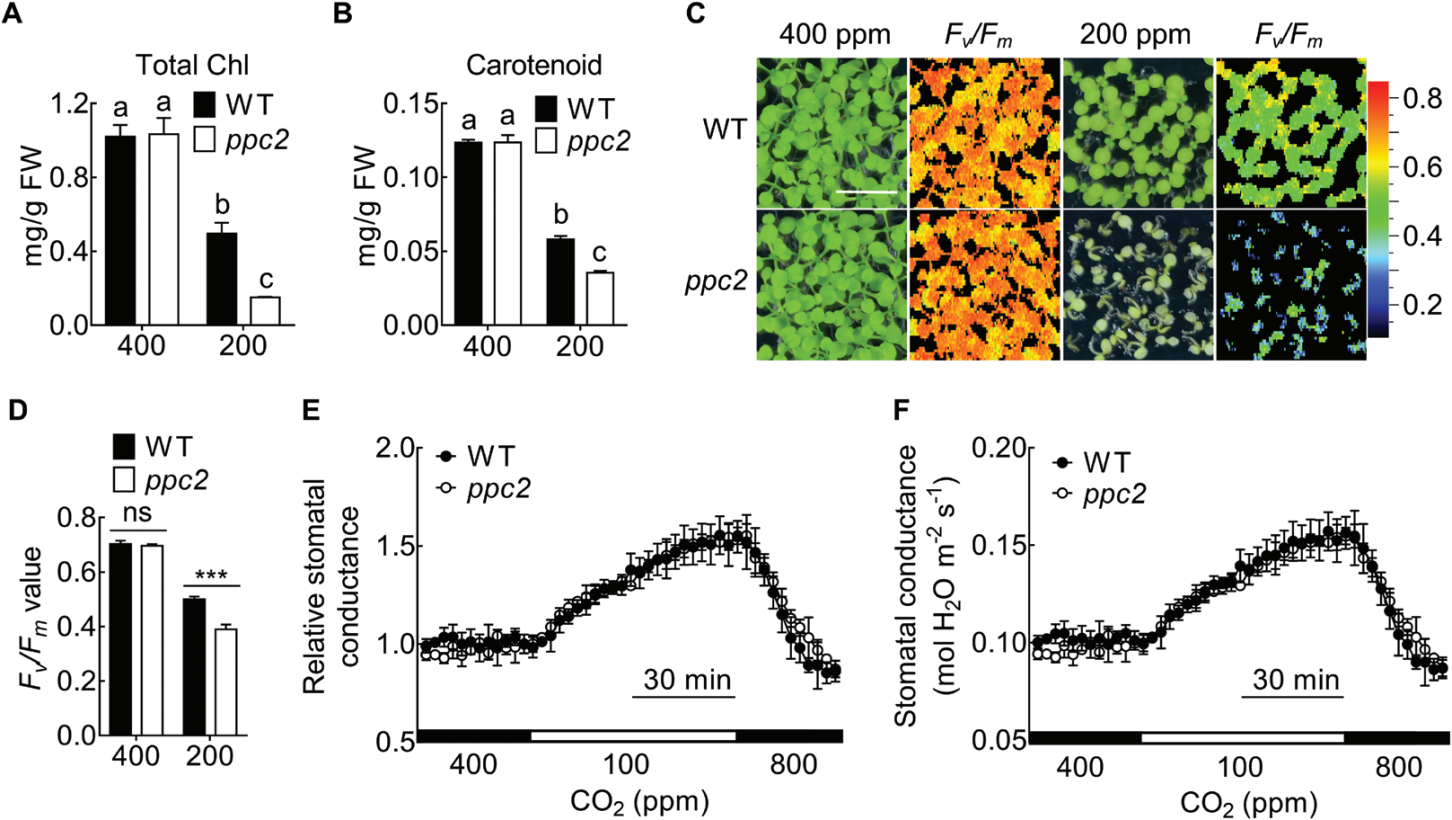
Carbon assimilation in the *ppc2* mutant under low CO_2_ conditions. (A, B) Chlorophyll content (A) and carotenoid content (B) in 15-day-old seedlings of the wild type (WT) and *ppc2* mutant at 400 ppm and 200 ppm CO_2_. Data shown are mean ±SEM (*n*=3). (C) *F*_v_*/F*_m_ was monitored by Closed FluorCam FC800 in WT and the *ppc2* mutant seedlings grown under 400 ppm or 200 ppm CO_2_ conditions for 15 d. (D) *F*_v_*/F*_m_ value comparison between the WT and *ppc2* mutant. Data shown are mean ±SEM (*n*=3). (E, F) Time-resolved stomatal conductance in response to CO_2_ changes in WT and the *ppc2* mutant plants. (E) Data shown in (F) were normalized. Different letters indicate significant differences using Tukey’s test at *P*≤0.05. Asterisks indicate significant differences between genotypes (***P<0.005 by Student’s *t*-test; ns, no significant difference).

Because *ppc2* plants were chlorotic under low CO_2_ conditions, we measured the chlorophyll and carotenoid contents in *ppc2* and Col-0 seedlings. Under ambient CO_2_ conditions, there were no significant differences in the total chlorophyll and carotenoid contents between *ppc2* and Col-0 (Fig. 3A, B). Under low CO_2_ conditions, both chlorophyll and carotenoid contents were greatly reduced in *ppc2*. We next determined the maximum quantum yield of PSII (*F*_v_*/F*_m_) in *ppc2* and Col-0 seedlings grown at 200 ppm and 400 ppm CO_2_ using the chlorophyll florescence detector FluorCam FC800. Under ambient CO_2_ conditions, there was no difference in *F*_v_*/F*_m_ between Col-0 and *ppc2*. Low CO_2_ led to obvious *F*_v_*/F*_m_ decreases in both Col-0 and *ppc2*, but the decrease was more remarkable in *ppc2* than in Col-0 (Fig. 3C, D). These results indicated the involvement of *PPC2* in photosynthesis regulation under low CO_2_ conditions.

Because *PPC2* was highly expressed in guard cells (Fig. 2C), it was necessary to clarify whether the reduced carbon assimilation rate under low CO_2_ conditions was induced by compromised low CO_2_-induced stomatal opening. The CO_2_ shift from 400 ppm to 100 ppm triggered a dramatic increase in stomatal conductance in both Col-0 and *ppc2* mutant plants. However, there were no significant differences in stomatal response to low CO_2_ between the *ppc2* mutant and Col-0 (Fig. 3E, F), indicating a minor role for *PPC2* in regulating stomatal opening. This also suggested that the reduced carbon assimilation in *ppc2* at low CO_2_ conditions was not caused by less CO_2_ uptake, but by defects in CO_2_ utilization.

### *ppc2* mutant seedlings showed growth arrest under mild drought stress conditions

**Fig. 4.**
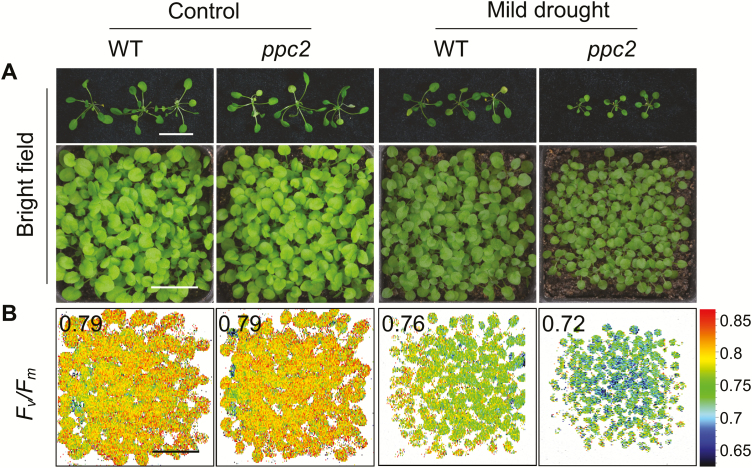
Growth arrest phenotype of the *ppc2* mutant seedlings under mild drought stress conditions. (A) Morphological phenotype and (B) *F*_v_*/F*_m_ monitored by Closed FluorCam FC800 of 10-day-old WT and *ppc2* mutant seedlings grown under normal and mild drought stress conditions for 10 d. Each pot had 64 seedlings. Scale bar=2 cm in (A) and (B).

Drought stress promotes stomatal closure, and thus decreases plant CO_2_ uptake and *C*_i_ (Parry *et al.*, 2013). We then determined whether *ppc2* had phenotypes under water shortage similar to those under low CO_2_ conditions. When plant watering was reduced, *ppc2* exhibited growth arrest, while under normal growth conditions no significant differences were observed between *ppc2* and Col-0 (Fig. 4A). Leaf temperature is an indicator of stomatal status (Jones, 1999). We found that drought stress increased leaf temperature in both *ppc2* and Col-0 compared with normal growth conditions, but no significant temperature differences between them were found under both normal and drought stress conditions (Supplementary Fig. S4). These results further support that drought stress promotes stomatal closure, and that *ppc2* plants retain normal stomatal responses. Furthermore, the *ppc2* mutant exhibited a reduced *F*_v_*/F*_m_ value under mild drought stress conditions (Fig. 4B), similar to low CO_2_ conditions. These results further suggest that *PPC2* is required for CO_2_ utilization and plant growth under low CO_2_ conditions caused by stresses such as drought.

### Application of exogenous sucrose or malate greatly rescued *ppc2* seedling growth arrest under low CO_2_ conditions

**Fig. 5.**
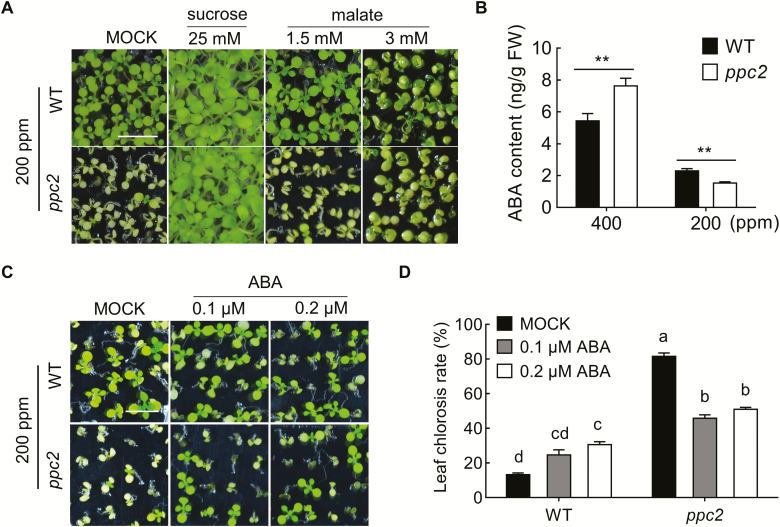
Exogenous ABA partially rescues *ppc2* seedling growth arrest under low CO_2_ conditions. (A) Phenotype of 15-day-old seedlings of wild-type (WT) and the *ppc2* mutant under 200 ppm CO_2_ conditions on sucrose-free 1/2 MS medium supplemented with mock (ddH_2_O), 25 mM sucrose, 1.5 mM malate, or 3 mM malate. Each replicate has at least 60 seedlings. (B) ABA contents in WT and the *ppc2* mutant seedlings grown under different CO_2_ concentrations. Data shown are mean ±SEM (*n*=3). (C) Phenotype and (D) statistical analysis of chlorosis rate of 15-day-old WT and the *ppc2* mutant seedlings grown at 200 ppm CO_2_ on sucrose-free 1/2 MS medium with mock (ddH_2_O), 0.1 μM ABA, and 0.2 μM ABA, respectively. Data shown are mean ±SEM (*n*=4). Each replicate has at least 60 seedlings. Asterisks indicate significant differences between genotypes (**P<0.01 by Student’s *t*-test). Different letters indicate significant differences using Tukey’s test at *P*≤0.05. Scale bar=1 cm in (A) and (C).

Considering the reduced photosynthetic carbohydrate accumulation and carbon assimilation in the *ppc2* mutant under low CO_2_ conditions, we further determined whether sucrose application could rescue the retarded growth of *ppc2* under low CO_2_ conditions. Interestingly, exogenous sucrose (25 mM) treatment completely rescued the seedling growth retardation and cotyledon chlorosis in *ppc2* under low CO_2_ conditions (Fig. 5A).

PEPC catalyzes oxaloacetate (OAA) synthesis from PEP and HCO_3_^−^. OAA is then rapidly converted into malate by malate dehydrogenase. Our primary metabolic analysis showed that *PPC2* mutation led to a decrease in malate (Supplementary Fig. S2B), particularly under low CO_2_ conditions. We then explored whether the *ppc2* mutant growth arrest phenotype under low CO_2_ conditions was caused by, or at least contributed by, malate defect. Application of 1.5 mM malate did not have any impact on the growth of either *ppc2* or Col-0, and also could not eliminate the *ppc2* cotyledon chlorosis phenotype (Fig. 5A). However, 3 mM malate greatly relieved the *ppc2* growth arrest phenotype compared with controls under our growth conditions. These results support *PPC2* functioning in both carbon assimilation and metabolic pathway.

### Exogenous ABA partially rescued *ppc2* seedling growth arrest under low CO_2_ conditions

ABA is synthesized from C_40_ oxygenated carotenoids (Ruiz-Sola and Rodriguez-Concepcion, 2012). The carotenoid content was noticeably reduced in *ppc2* under low CO_2_ concentrations (Fig. 3B). To determine whether ABA biosynthesis is blocked in *ppc2* under low CO_2_ conditions, we quantified the ABA level in *ppc2* and Col-0 seedlings grown under low and ambient CO_2_ conditions by UFLC-ESI-MS (Liu *et al.*, 2012). Interestingly, the ABA content in the *ppc2* mutant was greatly reduced under low CO_2_ conditions, but increased under ambient CO_2_ conditions compared with that in Col-0 (Fig. 5B). It can be speculated that the decrease in ABA level might be a cause of the *ppc2* growth arrest under low CO_2_ conditions.

To prove this, exogenous ABA (0.1 μM or 0.2 μM) was added in sucrose-free 1/2 MS medium plates to observe the growth performance of Col-0 and *ppc2* under low CO_2_ conditions. ABA treatment inhibited the growth and increased the chlorotic seedling ratio in Col-0, while, in the *ppc2* mutant, ABA treatment largely recovered the seedling growth arrest phenotype (Fig. 5C, D). Collectively, our results demonstrate that *PPC2* mutation affects ABA biosynthesis, and the *ppc2* seedling growth arrest phenotype is at least partly due to the decrease in ABA level.

### *ABI5* overexpression rescued *ppc2* seedling growth arrest under low CO_2_ conditions

**Fig. 6.**
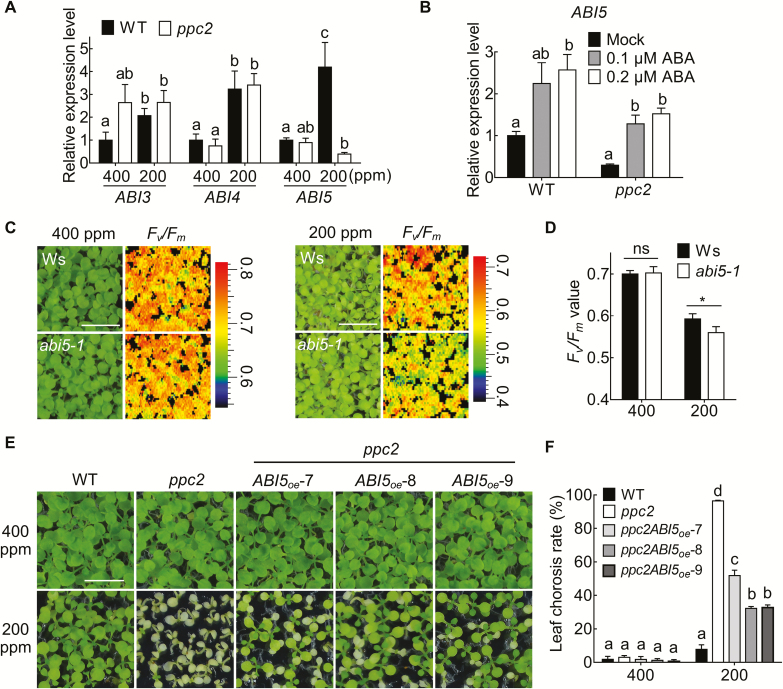
*ABI5* overexpression greatly rescues *ppc2* growth arrest under low CO_2_ conditions. (A) Expression levels of *ABI3*, *ABI4*, and *ABI5* relative to that of *EF1α* (AT5G60390) in the wild type (WT) and the *ppc2* mutant grown on sucrose-free 1/2 MS medium at 400 ppm or 200 ppm CO_2_ for 15 d. Data are shown as mean ±SEM (*n*=3). (B) *ABI5* expression levels in *ppc2* and WT seedlings treated with mock (ddH_2_O), 0.1 μM ABA, and 0.2 μM ABA at 200 ppm, respectively. Data shown are mean ±SEM (*n*=3). Different letters indicate significant differences using Tukey’s test at *P*≤0.05. (C) *F*_v_*/F*_m_ monitored by Closed FluorCam FC800 in Ws and the *abi5-1* mutant seedlings grown at 400 ppm or 200 ppm CO_2_ for 15 d. (D) Maximum photosynthetic yields (*F*_v_*/F*_m_) of Ws and the *abi5-1* mutant at different CO_2_ concentrations. Data shown are mean ±SEM (*n*=3). Asterisks indicate significant differences between genotypes (*P<0.05 by Student’s *t*-test; ns, no significant difference). (E) Phenotype and (F) statistical analysis of the chlorosis rate of the WT, the *ppc2* mutant, and *ABI5*-overexpressing *ppc2* transgenic lines (*ppc2ABI5*_oe_-7, *ppc2ABI5*_oe_-8, and *ppc2ABI5*_oe_-9). Data shown are mean ±SEM (*n*=3). Each replicate has at least 60 seedlings. Different letters indicate significant differences using Tukey’s test at *P*≤0.05. Scale bar=1 cm in (C) and (E).

It has been reported that *ABI3*, *ABI4*, and *ABI5* (the downstream targets of the ABA signaling pathway) are required for ABA modulation of seed germination and post-germination development (Finkelstein and Lynch, 2000; Lopez-Molina *et al.*, 2001). To determine whether ABI transcription factors function in *ppc2* seedling growth arrest under low CO_2_ conditions, we first determined their expression levels by qPCR. Low CO_2_ treatment induced *ABI3* and *ABI4* expression in both Col-0 and the *ppc2* mutant. However, *ABI5* induction by low CO_2_ conditions was greatly suppressed in *ppc2* (Fig. 6A). Furthermore, exogenous ABA rescued *ABI5* expression in the *ppc2* mutant under low CO_2_ conditions (Fig. 6B). These results indicated that the decrease in *ABI5* expression might be a major cause of *ppc2* growth arrest under low CO_2_ conditions. To confirm this possibility and reveal the role of *ABI5* in seedling growth under low CO_2_ conditions, we evaluated the growth performance of the *abi5-1* mutant and wild-type Wassileskija (Ws) plants under low CO_2_ conditions on sucrose-free medium. No obvious morphological differences were observed between Ws and *abi5-1* seedlings (Fig. 6C). However, the *F*_v_/*F*_m_ value of *abi5-1* was significantly less than that of Ws under low CO_2_ conditions (Fig. 6D), indicating that *ABI5* mutation led to reduction of maximum quantum efficiency of PSII under low CO_2_ conditions. We further overexpressed *ABI5* in *ppc2* driven by the constitutive CaMV 35S promoter. *ABI5* overexpression greatly rescued the seedling growth arrest phenotype of three randomly selected *ppc2* transgenic lines under low CO_2_ conditions (Fig. 6E, F; Supplementary Fig. S3).

### Photorespiratory intermediates were increased in *ppc2* under low CO_2_ conditions

**Fig. 7.**
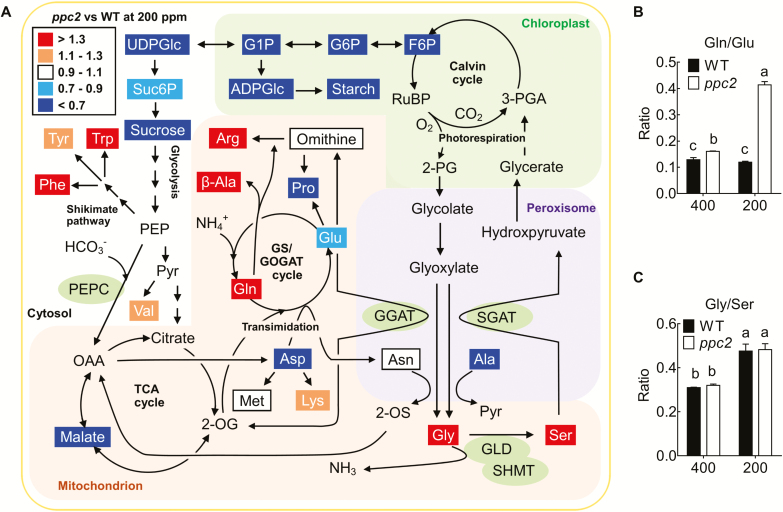
Metabolite analysis of the *ppc2* mutant and WT under low CO_2_ conditions. (A) Differences in leaf metabolite levels between the *ppc2* mutant and wild-type (WT) seedlings under 200 ppm CO_2_ conditions. PEPC, phosphoenolpyruvate carboxylase. GGAT, glutamate:glyoxylate aminotransferase. GLD, glycine decarboxylase, including GLDP, GLDT, and GLDH. SHMT, serine hydroxymethyltransferase. SGAT, serine:glyoxylate aminotransferase. RuBP, ribulose-1,5-disphosphate. F6P, fructose 6-phosphate. G6P, glucose 6-phosphate. G1P, glucose 1-phosphate. UDPGlc, UDP-glucose. ADPGlc, ADP-glucose. Suc6P, sucrose 6-phosphate. Pyr, pyruvate. 2-OS, 2-oxosuccinamate. OAA, oxaloacetate. PEP, phosphoenlpyruvate. (B) Glutamine to glutamate ratio in 15-day-old WT and *ppc2* seedlings at different CO_2_ concentrations. Data shown are mean ±SEM (*n*=3). (C) Ratio of glycine to serine ratio in 15-day-old WT and *ppc2* seedlings at different CO_2_ concentrations. Data shown are mean ±SEM (*n*=3). Different letters indicate significant differences using Tukey’s test at *P*≤0.05.

It has been suggested that PEPC activity is linked to photorespiration by supplying malate to the TCA cycle to sustain glutamate and glutamine metabolism (Masumoto *et al.*, 2010; Shi *et al.*, 2015). We then determined amino acid contents in *ppc2* and Col-0 under both ambient and low CO_2_ conditions. Under low CO_2_ conditions, the *ppc2* mutant exhibited reduced glutamate but increased glutamine, leading to increases in the glutamine to glutamate ratio (Fig. 7A, B) and the levels of β-alanine and arginine, with decreases in the levels of alanine, asparatic acid, and proline (Supplementary Fig. S5). Glycine and serine levels have been recognized as indicators of carbon flux through photorespiration, and a higher glycine/serine ratio indicates higher photorespiration (Novitskaya *et al.*, 2002). Low CO_2_ concentrations would increase photorespiration. The *ppc2* mutant had greater levels of the photorespiratory intermediates glycine and serine under photorespiratory (low CO_2_, 21% O_2_) conditions (Fig. 7A), and greater glycine levels at 400 ppm CO_2_ (Supplementary Fig. S5), consistent with the reduced photosynthesis and growth arrest phenotypes of *ppc2* under low CO_2_ conditions (Figs 3E, 5A). We found that low CO_2_ (photorespiratory conditions) triggered similar increases in the glycine/serine ratio in both *ppc2* and Col-0 compared with ambient CO_2_ conditions (Fig. 7C). Under ambient CO_2_ conditions, *PPC2* mutation did not show significant effects on amino acid and organic acid contents, and only glycine, valine, and tyrosine were slightly increased (Supplementary Figs S2B, S5). These results suggest that *PPC2* functions in both primary metabolism and photorespiratory metabolism under low CO_2_ conditions through modulation of the carbon–nitrogen balance.

### ABI5 regulated photorespiratory enzyme expression levels

**Fig. 8.**
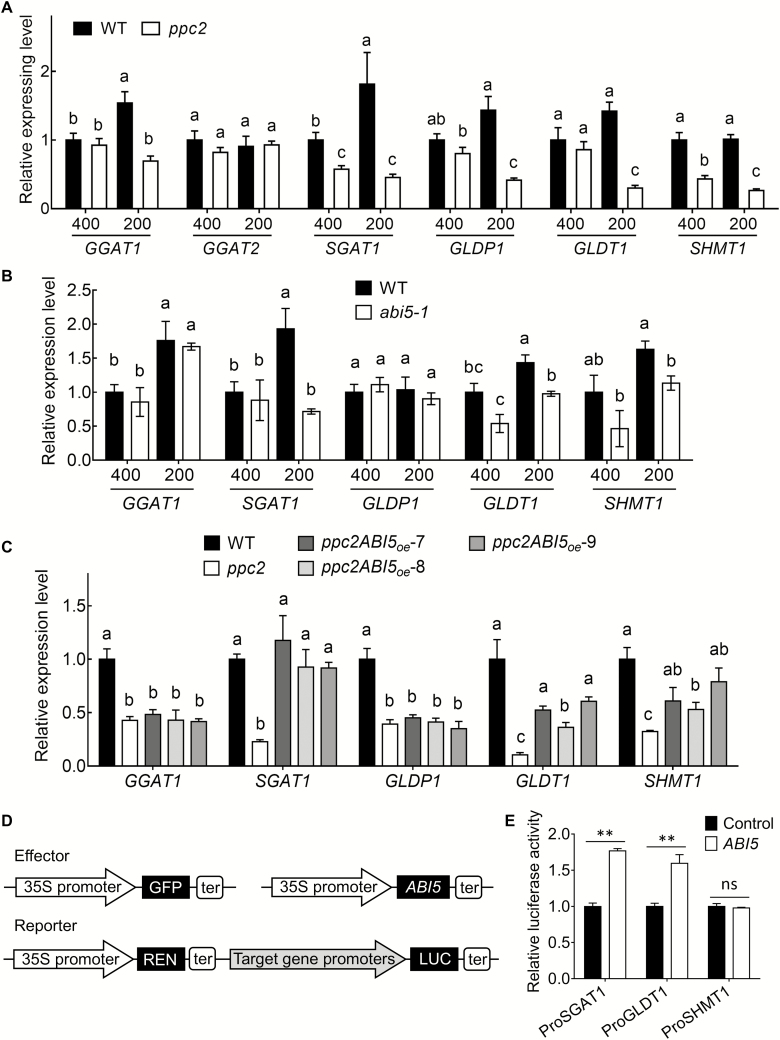
ABI5 regulates the expression levels of major photorespiratory enzymes related to glycine and serine synthesis and metabolism. (A) Expression levels of photorespiratory enzyme genes in wild-type (WT) and the *ppc2* mutant leaves. RNAs were extracted from the leaves of 15-day-old seedlings. *EF1α* (AT5G60390) was used as an internal control. Data shown are mean ±SEM (*n*=3). (B) Expression levels of photorespiratory genes in WT, *ppc2* mutant, and *ABI5-*overexpressing *ppc2* plants (*ppc2ABI5*_oe_-7, *ppc2ABI5*_oe_-8, and *ppc2ABI5*_oe_-9). RNAs were extracted from the leaves of 15-day-old seedlings at different CO_2_ concentrations. *EF1α* (AT5G60390) was used an internal control. Data shown are mean ±SEM (*n*=3). Different letters indicate significant differences using Tukey’s test at *P*≤0.05. (B) Expression levels of photorespiratory genes in the WT (Ws) and *abi5-1* mutant leaves. RNAs were extracted from the leaves of 15-day-old seedlings. *EF1α* (AT5G60390) was used as an internal control. Data shown are mean ±SEM (*n*=3). (D) A schematic representation of the dual-luciferase reporter system. *ABI5* or GFP (control) driven by CaMV 35S as effector was co-transformed with reporters, *REN* (Renilla luciferase) driven by 35S and *LUC* (firefly luciferase) driven by the promoter regions of photorespiratory genes. (E) ABI5 activation of *SGAT1*, *GLDT1*, and *SHMT1* by dual-luciferase reporter assays. LUC values were normalized to those of REN. Data shown are mean ±SEM (*n*=3). Asterisks indicate significant differences between genotypes (**P<0.01 by Student’s *t*-test; ns, no significant difference).

In the photorespiratory pathway, GGAT (glutamate:glyoxylate aminotransferase) transfers -NH_3_^+^ from glutamate into glyoxylate to generate glycine, and SGAT1 (serine:glyoxylate aminotransferase) transfers -NH_3_^+^ from serine, alanine, and asparagine into glyoxylate to produce glycine. GLDP1 and GLDT1 are components of the glycine decarboxylase complex, which catalyzes glycine into CH_2_-THF. To further explore whether the greater serine and glycine levels in *ppc2* under low CO_2_ conditions were caused by enzyme expression changes involved in glycine and serine synthesis and metabolism during photorespiration, we checked the expression levels of *GGAT1*, *GGAT2*, *SGAT1*, *GLDP1*, *GLDT1*, and *SHMT1* (Peterhansel *et al.*, 2010) in *ppc2* under both low and ambient CO_2_ conditions. Except for *GGAT2*, the expression levels were significantly reduced in *ppc2* under low CO_2_ conditions (Fig. 8A). Among them, *GGAT1* and *SGAT1* were slightly induced by low CO_2_ in Col-0 (Fig. 8A). *SHMT1* expression in *ppc2* was significantly reduced under both ambient and low CO_2_ conditions (Fig. 8A).

ABI5 is a transcription factor that directly binds to the promoter regions of its targets to activate their expression. *ABI5* expression was reduced in *ppc2*, and *ABI5* overexpression rescued the *ppc2* growth arrest phenotype under photorespiratory conditions. Thus, we speculated that ABI5 might regulate enzyme expression levels that function in glycine and serine metabolism, such as *GGAT1*, *SGAT1*, *GLDP1*, *GLDT1*, and *SHMT1*. Interestingly, there were several ABREs in their promoter regions (Supplementary Fig. S6). *SGAT1*, *GLDT1*, and *SHMT1* expression was reduced in *abi5-1* under low CO_2_ conditions, and their expression in *ppc2* was completely or greatly recovered by *ABI5* overexpression (Fig. 8B, C), suggesting that these three genes may be the targets of ABI5. We then performed dual-luciferase assays to determine whether ABI5 activates the promoters of *SGAT1*, *GLDT1*, and *SHMT1* that drive LUC expression in Arabidopsis protoplasts. ABI5 expression greatly activated *SGAT1* and *GLDT1* expression, but could not activate *SHMT1* expression (Fig. 8D, E), demonstrating that ABI5 regulates photorespiration by modulating photorespiratory enzyme expression levels, and *SGAT1* and *GLDT1* are the direct targets, and *SHMT1* is an ABI5 indirect target.

### Carbon assimilation was reduced in *ppc2* mature plants under photorespiratory conditions

**Fig. 9.**
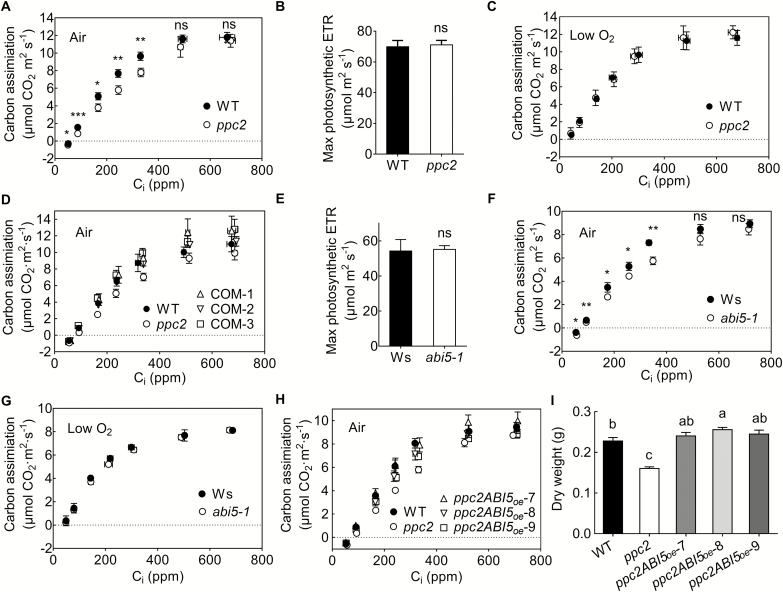
Carbon assimilation of the *abi5-1* mutant and the effect of *ABI5* overexpression on carbon assimilation of the *ppc2* mutant under low CO_2_ conditions. (A, C) *A*–*C*_i_ curves of 30-day-old wild type (WT) and the *ppc2* mutant plants under different CO_2_ conditions balanced with air (A) or low oxygen conditions (C). The light intensity for the measurement was set at 2000 μmol m^−2^ s^−1^. Data shown are mean ±SEM (*n*=3). (B) Maximum photosynthetic electron transport rate (ETR) of 30-day-old WT and *ppc2* mature plants. Data shown are mean ±SEM (*n*=5). (D) *PPC2* expression complements the reduced carbon assimilation in the *ppc2* rosette leaves grown under 400 ppm CO_2_ conditions. COM-1, COM-2, and COM-3 are *PPC2* complementary lines. Data shown are mean ±SEM (*n*=3). (E) Maximum photosynthetic ETR of 30-day-old Ws and *abi5-1* mutant plants. Data shown are mean ±SEM (*n*=5). (F, G) *A*–*C*_i_ curves of 30-day-old *abi5-1* and Ws under different CO_2_ conditions balanced with air (F) or low oxygen (G). Data shown are mean ±SEM (*n*=3). (H) *ABI5* overexpression complements the reduced carbon assimilation of *ppc2* plants grown under 400 ppm CO_2_ conditions. Data shown are mean ±SEM (*n*=3). (I) Dry weight of WT, *ppc2*, and *ABI5-*overexpressing *ppc2* transgenic lines (*ppc2ABI5*_oe-7_, *ppc2ABI5*_oe-8_, and *ppc2ABI5*_oe-9_) grown under ambient CO_2_ conditions. Data shown are mean ±SEM (*n*=6). Asterisks indicate significant differences between genotypes (*P<0.05; **P<0.01; ***P<0.005 by Student’s *t*-test; ns, no significant difference). Different letters indicate significant differences using Tukey’s test at *P*≤0.05.

Recently, a potential role for PEPC in C_3_ plant metabolism under high photorespiratory (low CO_2_, 21% O_2_) conditions was proposed (Abadie and Tcherkez, 2019; Tcherkez and Limami, 2019). Here, we also found that PPC2 is involved in seedling development by modulating photorespiratory metabolism under low CO_2_ conditions. To further investigate *PPC2* function under photorespiratory conditions, we determined the CO_2_ assimilation rate under different *C*_i_ conditions in *ppc2* and Col-0 mature leaves under ambient air conditions. Compared with Col-0, the *ppc2* mutant exhibited a lower CO_2_ assimilation rate under low CO_2_ (50–400 ppm) concentrations, and displayed a similar CO_2_ assimilation rate under high CO_2_ (400–800 ppm) concentrations as observed in the *A*–*C*_i_ curve (Fig. 9A). In addition, no significant difference was observed in the maximum photosynthetic electron transport rates (ETRs) calculated from the *A*–*C*_i_ curves between Col-0 and the *ppc2* mutant (Fig. 9B), indicating that *ppc2* mutation did not alter the photosynthetic capacity. The reduction in the initial *A*–*C*_i_ curve was recovered when the measurements were performed under very low oxygen conditions that restricted photorespiration (Fig. 9C). These results indicated that the altered CO_2_ assimilation of *ppc2* under low CO_2_ conditions was associated with the simultaneous photorespiratory CO_2_ loss. Moreover, *PPC2* expression rescued the reduced photosynthetic rate in response to low C_i_ conditions in *ppc2* (Fig. 9D). Together with the fact that PEPC is an important enzyme in the glycolytic pathway that links with photorespiration and respiration in plants, our results suggest that *PPC2* is involved in photorespiration under relatively low CO_2_ conditions. The phenotype of *ppc2* is at least partially due to the reduction of net carbon assimilation, which may result from the low capacity to utilize the photorespiratory metabolites under relatively low CO_2_ conditions when *PPC2* is mutated.

### *ABI5* overexpression rescued the reduced CO_2_ assimilation in mature *ppc2* plants under photorespiratory conditions

We also determined the *A*–*C*_i_ curves of 30-day-old *abi5-1* plants under ambient air and low oxygen conditions. Under ambient air conditions, *ABI5* mutation greatly reduced the CO_2_ assimilation rate under low CO_2_ concentrations (50–400 ppm), but not under high CO_2_ concentrations (600–800 ppm) compared with Ws (Fig. 9F). The maximum photosynthetic ETRs inferred from the *A*–*C*_i_ curves were similar between Ws and *abi5-1* under ambient air conditions (Fig. 9E). Under low oxygen conditions, the slopes of the *A*–*C*_i_ curve showed no significant difference between *abi5-1* and Ws (Fig. 9G). These results demonstrate that *ABI5* is involved in photorespiratory metabolism.

We next determined the *A*–*C*_i_ curves of *ABI5*-overexpressing *ppc2* plants grown under ambient CO_2_ conditions. The reduced CO_2_ assimilation of *ppc2* at low CO_2_ was completely rescued by *ABI5* overexpression (Fig. 9H). Consistent with this, *ABI5* overexpression restored and even slightly increased the *ppc2* mutant reduced dry weight biomass under ambient conditions compared with Col-0 (Fig. 9I).

## Discussion

### PPC2 is essential for plant acclimation to low CO_2_

CO_2_ is the major source for photosynthesis and is pivotal for plant growth. High CO_2_ usually increases plant growth and reproduction, whereas low CO_2_ decreases plant growth by changing some physiological responses (e.g. water use efficiency), reducing biomass production, and delaying development (Gerhart and Ward, 2010). However, the mechanisms underlying the effects of low CO_2_ concentrations on plants are still unclear. Here, we report that Arabidopsis *PPC2*, which encodes a PEPC involved in plant primary metabolism for production of C4-dicarboxylic acids, is essential for plant growth under low CO_2_ conditions. *ppc2* seedlings showed chlorosis and growth arrest under low CO_2_ (200 ppm) conditions (Fig. 1C, D), which could be rescued by *PPC2* expression (Fig. 1E, F). Moreover, there were no significant differences in the germination rate between *ppc2* and Col-0 under low CO_2_ conditions (Supplementary Fig. S1), suggesting that the phenotypes of the *ppc2* mutant occurred at the seedling development stage. Compared with Col-0, *ppc2* mutant seedlings accumulated less photosynthetic carbohydrates in leaves, such as sucrose, starch, and their upstream precursors (Supplementary Fig. S2), and exogenous sucrose or malate application greatly recovered the *ppc2* growth arrest phenotype (Fig. 5A). *PPC2* mutation greatly decreased the total leaf PEPC activity under low CO_2_ conditions (Fig. 2B; Supplementary Fig. S2B). Furthermore, among these three Arabidopsis plant-type PEPCs, only *PPC2* was induced by low CO_2_, consistent with the previous study (Li *et al.*, 2014), and *PPC1* or *PPC3* mutation did not affect plant growth at low CO_2_ (Figs 1C, 2A) (Li *et al.*, 2014; Shi *et al.*, 2015; Feria *et al.*, 2016). All these results suggest that PPC2 is essential for plant growth under low CO_2_ conditions, and is the major PEPC in Arabidopsis leaves.

### PPC2 participates in photorespiration by linking with primary metabolism under photorespiratory conditions

Recent studies in sunflower have shown that malate content closely correlates with photorespiration by metabolomics analysis, and C_3_ PEPC CO_2_ fixation increases under high photorespiratory conditions (low CO_2_, 21% O_2_) (Abadie *et al.*, 2017; Abadie and Tcherkez, 2019), indicating that non-photosynthetic PEPC may still contribute to photorespiration in C_3_ plants. Studies in Arabidopsis have reported the novel function of C_3_ PEPC in regulating the balance of carbon–nitrogen metabolism (Masumoto *et al.*, 2010; Shi *et al.*, 2015). However, the molecular mechanisms remain elusive. Here, we for the first time clarified the special role of *PPC2* in photorespiration by regulating the carbon–nitrogen balance under low CO_2_ conditions.

First, under photorespiratory conditions, PPC2 may regulate carbon–nitrogen balance. Glycolysis pathway metabolites and amino acid levels were greatly affected (Fig. 7A; Supplementary Figs S2B, S4), and the photorespiratory intermediates glycine and serine were significantly accumulated in the *ppc2* mutants (Fig. 7A; Supplementary Fig. S5), suggesting that PPC2 is involved in photorespiratory metabolism under low CO_2_ conditions. Glutamate, which plays a central signaling and metabolic role in regulating carbon and nitrogen assimilatory pathways (Forde and Lea, 2007), was decreased in *ppc2* under photorespiratory conditions. The increase in glutamine further reduced ammonium assimilation released by glycine decarboxylation in photorespiration, and thus probably contributed to glycine accumulation at low CO_2_. Exogenous malate application not only greatly rescued the growth arrest of *ppc2* under low CO_2_ conditions (Fig. 5A), but also reduced the high accumulation of glycine in the *ppc1/ppc2* double mutant (Shi *et al.*, 2015). These phenomena are in accordance with the previous report that malate dehydrogenase mutants exhibit an alteration in photorespiratory metabolism (Tomaz *et al.*, 2010). Because of *PPC2* mutation, PEP flux into the glycolysis pathway was reduced, while that into the shikimate pathway was increased under low CO_2_ conditions, leading to greater phenylalanine, tyrosine, and tryptophan levels (Fig. 7A; Supplementary Fig. S5). These results demonstrate that PPC2 controls carbon and nitrogen metabolism balance under low CO_2_ conditions.

Secondly, under photorespiratory conditions, *PPC2* affects the expression patterns of photorespiratory enzyme related to glycine and serine synthesis and metabolism. In the photorespiratory pathway, GGATs and SGAT1 transfer -NH_3_^+^ from glutamate and serine, respectively, into glyoxylate to synthesize glycine; GLDP1 and GLDT1 decarboxylate glycine into CH_2_-THF; and subsequently SHMT1 transfers the C1 moiety to another glycine to result in serine formation (Peterhansel *et al.*, 2010). Mutation of either *SGAT1* or *SHMT1* leads to serine and glycine accumulation (Somerville and Ogren, 1980; Kuhn *et al.*, 2013). Furthermore, glycine decarboxylase overexpression resulted in lower glycine and serine contents (Timm *et al.*, 2015). In agreement with the above results, *SHMT1*, *SGAT1*, *GLDP1*, and *GLDT1* expression was significantly reduced in *ppc2* under low CO_2_ conditions, which increased glycine and serine levels.

Thirdly, decreased CO_2_ assimilation in *ppc2* under low CO_2_ conditions was caused by increased photorespiratory carbon loss. The *ppc2* mutant exhibited decreased CO_2_ assimilation at relatively lower CO_2_ concentrations (50–400 ppm) (Fig. 9A). However, after reducing the O_2_ concentration to inhibit photorespiration, the reduced CO_2_ assimilation in *ppc2* under low CO_2_ conditions was recovered (Fig. 9C). Moreover, our results showed that the reduced carbon assimilation of *ppc2* during low CO_2_ conditions was not related to the stomatal status, because the stomatal conductance at the steady state and in response to the low CO_2_ shift remained normal in *ppc2* (Fig. 3E, F). In this sense, *PPC2* is specifically involved in photorespiration under low CO_2_ conditions (Fig. 9D).

### ABA regulates photorespiration under low CO_2_ conditions through ABI5

ABA induces gene expression and plays a prominent role in establishment of stress tolerance. However, little is known about the relationships of ABA to low CO_2_ stress and the simultaneous photorespiration. In this study, we found that ABA biosynthesis was heavily blocked under low CO_2_ conditions, possibly due to the decrease in accumulation of carotenoids (Fig. 3B), which are ABA biosynthesis precursors (Ruiz-Sola and Rodriguez-Concepcion, 2012). *PPC2* mutation further reduced ABA synthesis (Fig. 5B). Application of a small amount of ABA largely recovered *ppc2* growth (Fig. 5C, D), suggesting that low ABA concentration is required for stimulating photosynthesis and plant growth under low CO_2_ conditions. In addition, it has been reported that exogenous ABA induces the photorespiratory rate in barley by increasing GOX (glycolate oxidase) activity (Popova *et al.*, 1987). Our data also showed that high ABA treatment led to leaf chlorosis in Col-0 (Fig. 5C, D). These results suggest that a greater ABA concentration could reduce photosynthesis under low CO_2_ conditions, possibly by promoting carbon flux through the photorespiratory cycle.

ABI5 has been proposed as a key player in monitoring environmental conditions during seedling growth (Lopez-Molina *et al.*, 2001), and to function as an intermediate in ABA signaling to regulate seed germination and seedling growth. Our results show that *ABI5* plays a key role in seedling growth under photorespiratory conditions by regulating photorespiratory enzyme expression, and ABA regulates plant growth under low CO_2_ conditions through modulating *ABI5* expression. *abi5-1* seedlings grown at low CO_2_ showed lower *F*_v_/*F*_m_ values (Fig. 6C, D). CO_2_ assimilation in the mature *abi5-1* mutant was reduced under low CO_2_ conditions, and could be fully recovered by non-photorespiratory (low O_2_) conditions (Fig. 9F, G). In the *ppc2* plants, ABA synthesis was reduced and *ABI5* was repressed by low CO_2_ conditions, while ABA treatment greatly rescued *ABI5* expression and *ppc2* growth arrest (Figs 5D, 6B). Moreover, the expression levels of photorespiratory enzymes related to glycine and serine metabolism decreased in *ppc2* and *abi5-1* seedlings under low CO_2_ conditions (Fig. 8A, C). Further experiments showed that ABI5 may regulate *SGAT1* and *GLDT1* by directly binding to their promoters, and regulates *SHMT1* indirectly (Fig. 8E). *ABI5* overexpression rescued the expression of the above genes (Fig. 8C) and the reduction of dry weight biomass in *ppc2* mature plants (Fig. 9H, I).

In summary, our study reveals that *PPC2* is essential for plant acclimation to low CO_2_ and plays an important role in carbon assimilation via regulating carbon and nitrogen metabolism balance under photorespiratory low CO_2_ conditions. We also identified the important role of ABA in photorespiration and the novel function of *ABI5* in regulating expression of photorespiratory enzyme under low CO_2_ conditions. Our work demonstrates the key role of C_3_-PEPC PPC2 in photorespiration under low CO_2_ conditions, and may offer clues for future studies to understand the mechanism of C_3_ PEPCs in photosynthesis regulation and for potential application in crop improvement against photorespiration.

## Supplementary data

Supplementary data are available at *JXB* online.

Fig. S1. Seed germination of the wild type and *ppc* mutants.

Fig. S2. Photosynthetic carbohydrates were reduced in the *ppc2* mutant seedlings under low CO_2_ conditions.

Fig. S3. RT-PCR analysis of ABI5 expression level in WT, *ppc2*, and *ABI5-*overexpressing *ppc2* plants.

Fig. S4. Leaf temperature of WT and *ppc2* mutant seedlings by thermal imaging under normal and mild drought stress conditions.

Fig. S5. Amino acid contents in WT and *ppc2* mutant seedlings.

Fig. S6. Predicted ABRE *cis*-elements in the promoter regions of photorespiratory genes.

Table S1. List of primers used in this study.

eraa148_suppl_supplementary_Material

## Data Availability

All data generated or analyzed during this study are included in this article.
