# Supplementary material for: Involvement of abscisic acid, ABI5, and PPC2 in plant acclimation to low CO2
Source: J Exp Bot. 2020 Mar 24;71(14):4093–108. doi: 10.1093/jxb/eraa148 (PMC7337093; doi:10.1093/jxb/eraa148)
Supplement: eraa148_suppl_supplementary_Material [file eraa148_suppl_supplementary_material.pdf]

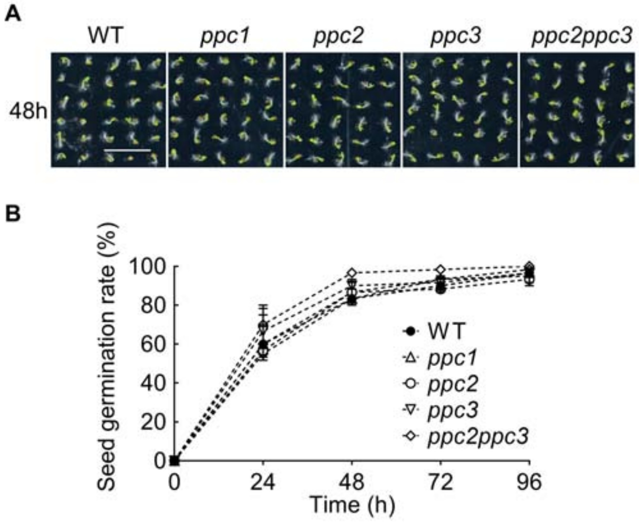

Fig. S1. Seeds germination of wild type and *ppc* mutants. (A) Seed germination of wild type (WT), *ppc1*, *ppc2*, *ppc3* and *ppc2ppc3* at 200 ppm CO_2 conditions at 48h. Bars = 1 cm. (B) Germination rates at different time course of WT, *ppc1*, *ppc2*, *ppc3* and *ppc2ppc3* mutants. Data shown are mean ± SEM (n = 3).

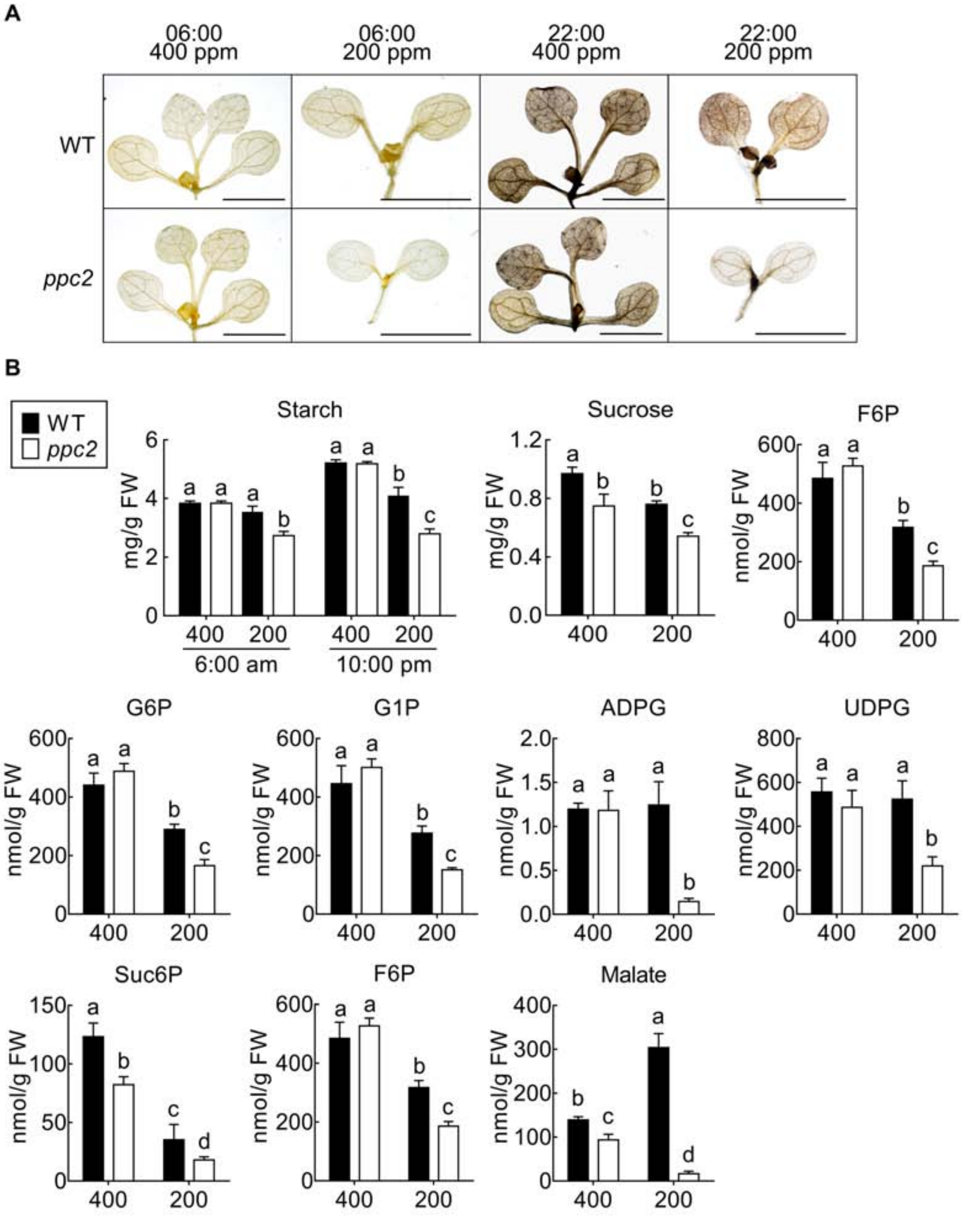

Fig. S2. Photosynthetic carbohydrates were reduced in *ppc2* mutant seedlings under low CO_2 conditions. (A) Iodine stained shoots of wild type (WT) and *ppc2* mutant seedlings. Shoots of 15-day-old WT and *ppc2* mutant seedlings which growing at different CO_2 conditions were harvested at 06:00 (end of the dark) and 22:00 (end of the light). Bar = 5 mm. (B) Changes in leaf metabolite content in WT and *ppc2* mutant. All the samples were harvested from 15-day-old WT and *ppc2* mutant seedlings at 200 ppm and 400 ppm CO_2 conditions. Samples of starch were harvested at 06:00 (end of the dark) and 22:00 (end of the light). Data shown are mean ± SEM (n = 3). G6P, glucose 6-phosphate. F6P, fructose 6-phosphate. G1P, glucose 1-phosphate. UDPGlc, UDP-glucose. ADPGlc, ADP-glucose. Suc6P, sucrose 6-phosphate. Data shown are mean ± SEM (n = 3). Different letters indicate significant difference using Tukey's test at $P \leq 0.05$.

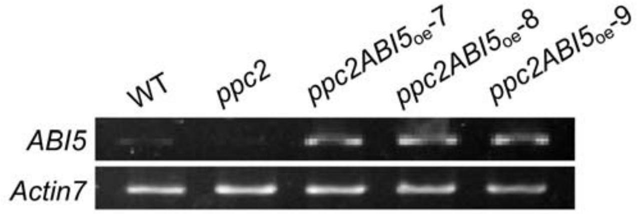

Fig. S3. RT-PCR analyses of *ABI5* expression level in WT, *ppc2* and *ABI5*-overexpressing *ppc2* plants. RNA was extracted from leaves of the 15-day-old seedlings. ACTIN7 (AT5G09810) was used as an equal loading control. *ppc2ABI5*$_{oe}$-7, *ppc2ABI5*$_{oe}$-8 and *ppc2ABI5*$_{oe}$-9 were *ABI5* expressing *ppc2* plants. WT, wild type.

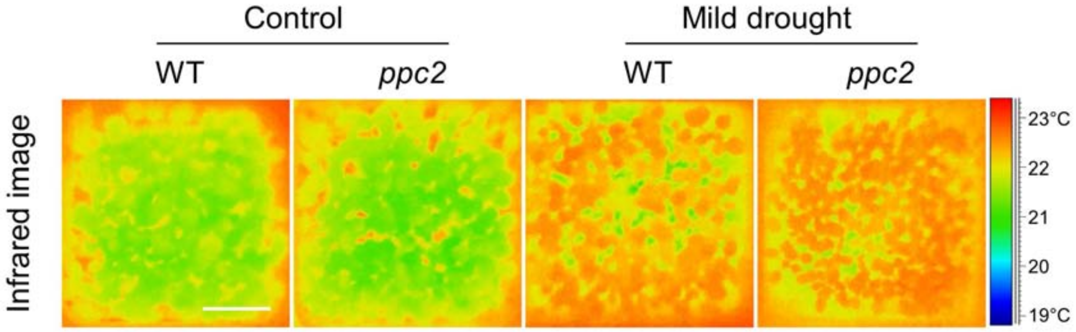

Fig. S4. Leaf temperature of WT and *ppc2* mutant seedlings by thermal imaging under normal and mild drought stress conditions. There were 64 seedlings of 10-day-old WT or *ppc2* mutant in each pot grown at drought stress conditions for 10 days. The control groups were normal watered. Bar = 2 cm.

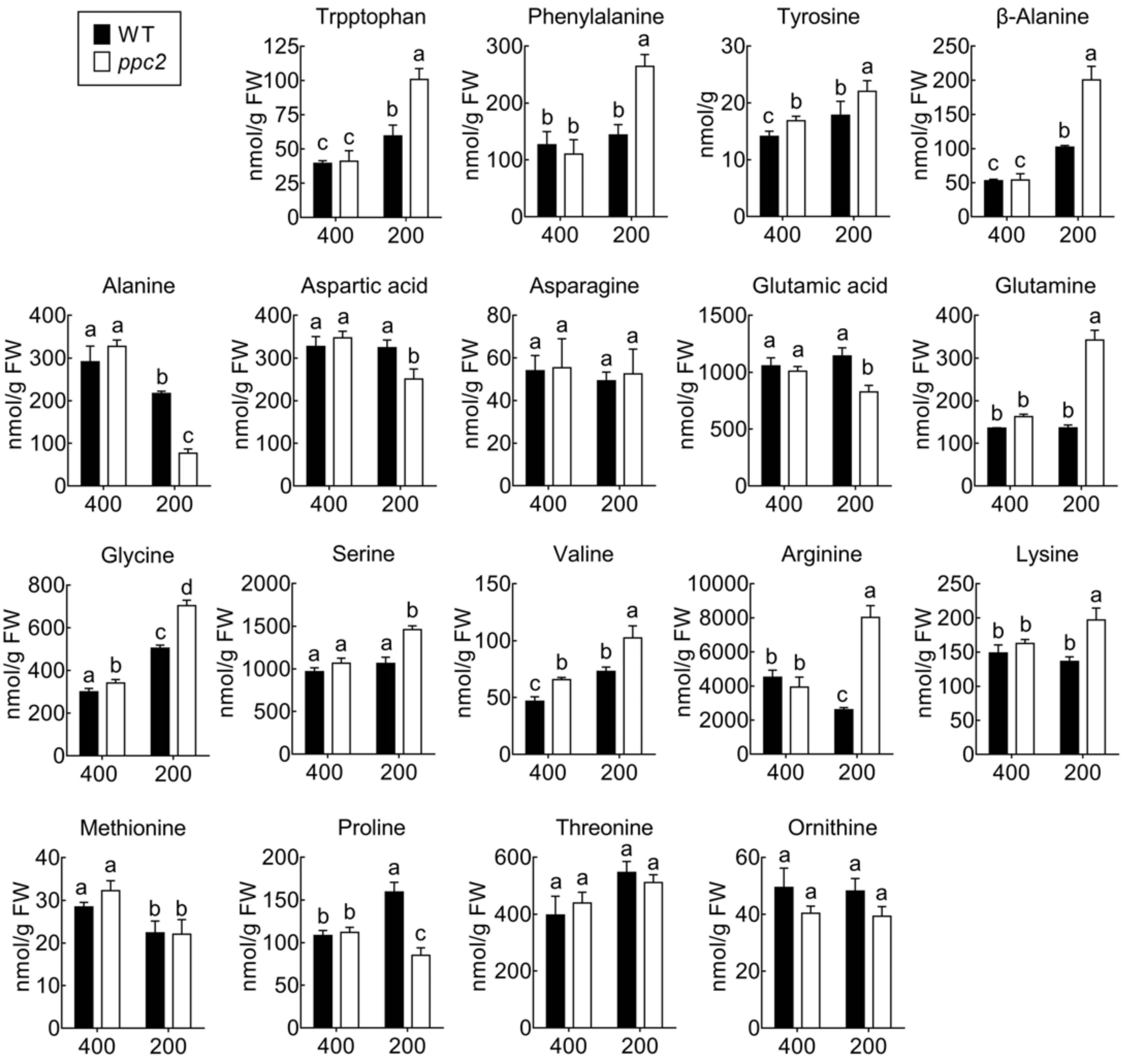

Fig. S5. Amino acid content in WT and *ppc2* mutant seedlings. Samples were harvested from 15-day-old wild type (WT) and *ppc2* mutant seedlings at 200 ppm and 400 ppm CO_2 conditions. Data shown are mean ± SEM (n = 3). Different letters indicate significant difference using Tukey's test at $P \leq 0.05$.

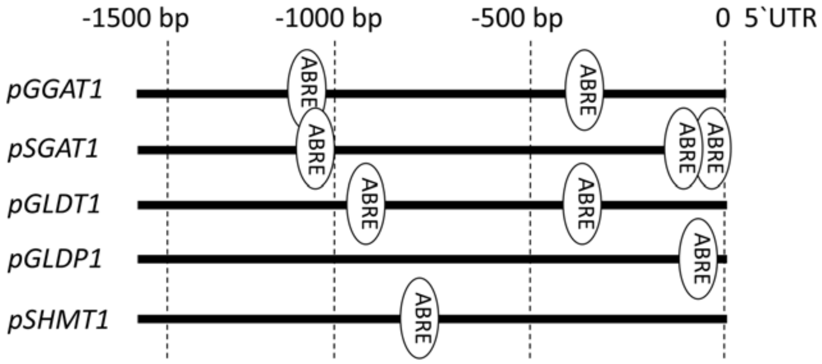

Fig. S6. Predicted ABRE cis-elements in the promoter regions of photorespiratory genes. 1.5 kb promoter sequence of photorespiratory enzyme genes were collected from the TAIR database and analyzed by the PlantCARE databases. The scales indicate the relative position of ABRE cis-element to the transcriptional start site.

Supplemental Table S1. List of primers used in this study.

Name	*Sequence (5`-3`)*
ppc1-gt-L	GACGTTTGCCACTTAGCTCAG
ppc1-gt-R	GAGCGGGAATGAGTTATTTCC
ppc2-gt-L	AAGGCCAAATGTTGACACTTG
ppc2-gt-R	GCAAGATGAAATGAGAGCAGG
ppc3-gt-L	CTGCATCTTTCCCTGAATCTG
ppc3-gt-R	GAACTGCACAGGAACTCAAGG
PPC1-RT-F	CGTGATTCTTACATTACAACTCT
PPC1-RT-R	CGATTCCGCTATCTCCTT
PPC2-RT-F	ATTGCCACTGAAGAATAC
PPC2-RT-R	CATCACATCATAGAACATAATAC
PPC3-RT-F	TGGCACAGTCGGAAGAGG
PPC3-RT-R	AGATTTCAACGGTGGATACATAA
ABI5-RT-F	TTTGGTCTGAGATACATAGAGGTG
ABI5-RT-R	GATTTCGGCAATTTCGGT
PPC2-OE-F	CGCACTAGTGCGATGGCTGCGAGAAATTTGGAG
PPC2-OE-R	GCGCCCGGGCGCTTAACCGGTGTTTTGCATACCAGC
ABI5-OE-F	GGGGACAAGTTTGTACAAAAAAGCAGGCTATGGTAACTAGAGAAACGAAGTTGAC
ABI5-OE-R	GGGGACCACTTTGTACAAGAAAGCTGGGTTTAGAGTGGACAACTCGGGTTC
Pro-PPC2-F	GAATTCGGCAATAATCAACCATGTATGC
Pro-PPC2-R	GGATCCGGTTTGGTTGATGCTTTTTCCTCTC
ABI3-qPCR-F	GATTACTCCGTTTGTGTT
ABI3-qPCR-R	GTTGTTGTTGTTGTTGTTA
ABI4-qPCR-F	AGAGATTACGGTTCCAAT
ABI4-qPCR-R	ATATTAAGGTAGGCACACT
ABI5-qPCR-F	GAAGAGGAAGCAACAGTATT
ABI5-qPCR-R	TCATCAATGTCCGCAATC
GGAT1-qPCR-F	TTCAAGAAGTTCAACGAC
GGAT1-qPCR-R	TCAGAAGAAGAAGTAATCAC
GGAT2-qPCR-F	TAGAGTCAGAGTCAGAGA
GGAT2-qPCR-R	TTAAGAGCACCAAGAAGA
SGAT1-qPCR-F	TGGTATGATTCTCGTCTT
SGAT1-qPCR-R	AATGTTGATGTGGTTGAG
GLDT1-qPCR-F	GGAACAAGATTGGAGAGA
GLDT1-qPCR-R	CTGACCTGACTTCACATA
GLDP1-qPCR-F	GGACAATGTATATGGAGAC
GLDP1-qPCR-R	GACAGAGCAGTAATCAAG
SHMT1-qPCR-F	CACACTATCACAGGACTA
SHMT1-qPCR-R	TACTCAGGACTTGTTCTT
Pro-SGAT1-F	CACGTCGACGTGTGGCTCTCTCCCTTTTATCTC
Pro-SGAT1utr-R	AAGCCATGGTTTTTCCTCTTTTCTTTTGGATCC
Pro-GLDT1-F	CACGTCGACACAAAACAAGCTTATATTCTATCCG
Pro-GLDT1utr-R	CATCCATGGCTTCTACACAACAAGTTTGATACAAGA
Pro-SHMT1N-F	AAGGTCGACTTGCGTGGAATTCGTATGAG
Pro-SHMT1utr-R	CATCCATGGTTTTCGCTAAACCTCTCTCTATCT
